# Supplementary material for: Histidine kinase MHZ1/OsHK1 interacts with ethylene receptors to regulate root growth in rice
Source: Nat Commun. 2020 Jan 24;11:518. doi: 10.1038/s41467-020-14313-0 (PMC6981129; doi:10.1038/s41467-020-14313-0)
Supplement: Supplementary file 1 — Supplementary Information [file 41467_2020_14313_MOESM1_ESM.pdf]

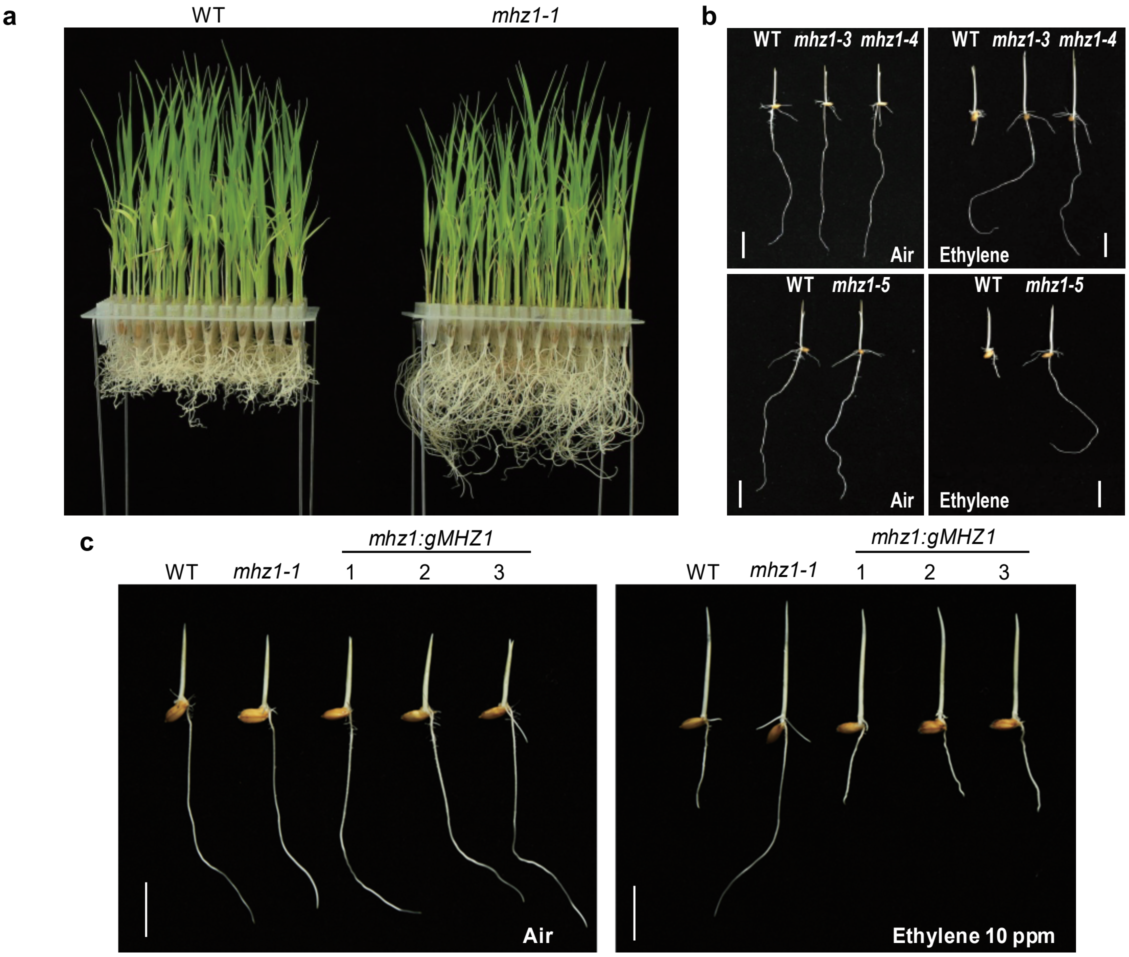

Supplementary Fig. 1 Ethylene insensitive phenotype of *mhz1-1* green seedlings and of different *mhz1* alleles. **a** Green seedlings of WT and *mhz1-1*. **b** The ethylene insensitive phenotype of *mhz1-3*, *mhz1-4* and *mhz1-5* etiolated seedlings. Rice seedlings were grown in darkness with air or 10 ppm ethylene treatment. Bars indicate 10 mm. **c** *MHZ1* genomic sequence (4.414 kb upstream of ATG codon, 5.623 kb genomic coding sequence and 1 kb downstream of stop codon) complemented the *mhz1-1* ethylene insensitive phenotype in segregated transgenic lines. Numbers indicate segregated WT-like plants from different transgenic lines. *gMHZ1* indicates *MHZ1* genomic sequence. Bars indicate 10 mm.

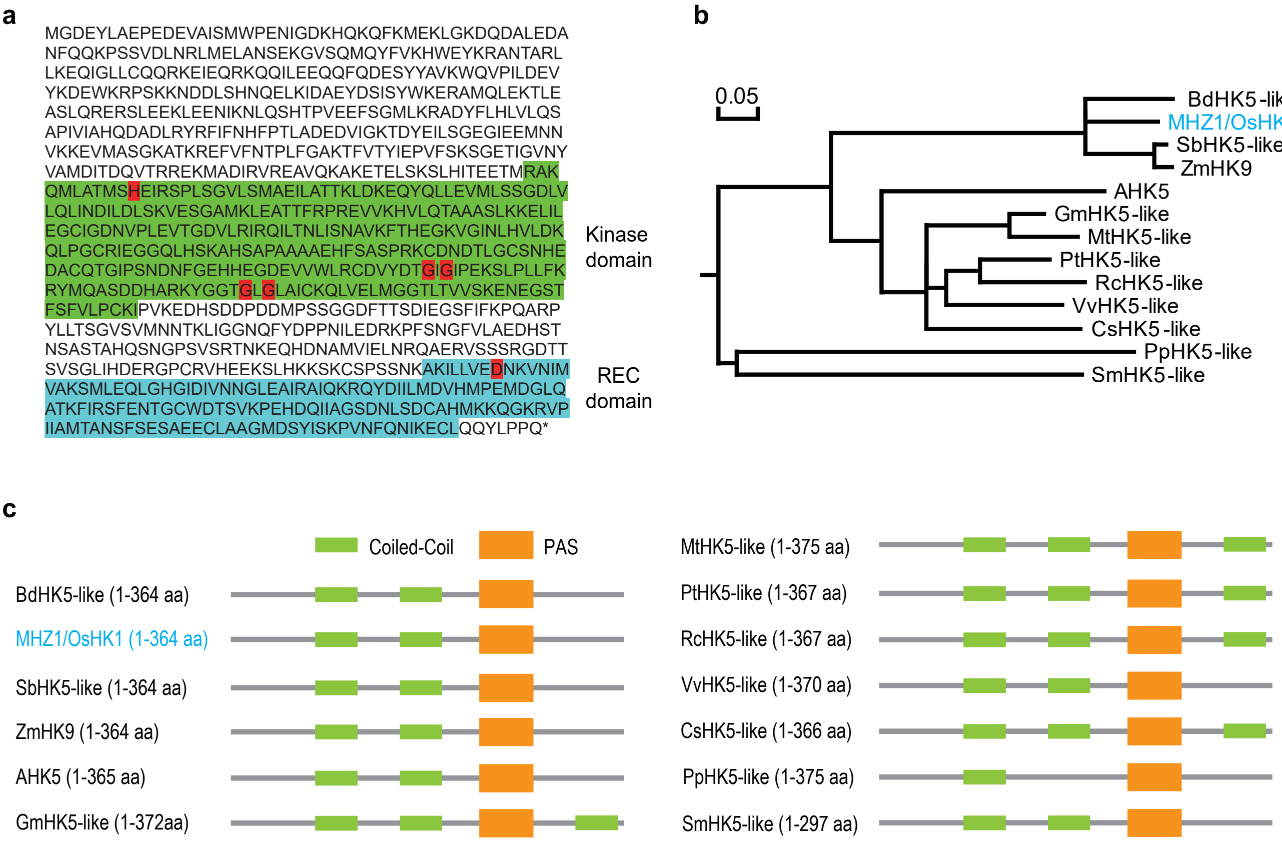

Supplementary Fig. 2 MHZ1/OsHK1 amino acid sequence and cluster analysis of MHZ1/OsHK1 and homologous sequences from other plant species. **a** MHZ1/OsHK1 amino acid sequence analysis. The kinase domain and REC domain were highlighted by different colors. Conserved histidine in kinase domain, glycine in G1 box and G2 box, aspartic acid in REC domain were marked out by red back color. **b** Cluster analysis for homologous proteins of MHZ1 from other plant species. The cluster analysis tree was generated by DNAMAN with maximum likelihood method. The rooted line distance indicates phylogenetic distance. AHK5, *Arabidopsis thaliana*, AT5G10720; BdHK5-like, *Brachypodium distachyon*, XP_003560378; CsHK5-like, *Cucumis sativus*, XP_004141443; GmHK5-like, *Glycine max*, XP_003544573; MtHK5-like, *Medicago truncatula*, XP_003588798; PpHK5-like, *Physcomitrella patens*, XP_001774097 ; PtHK5-like, *Populus trichocarpa*, XP_002324940; RcHK5-like, *Ricinus communis*, XP_002515371; SmHK5-like, *Selaginella moellendorffii*, XP_002985908; SbHK5-like, *Sorghum bicolor*, XP_002437388; VvHK5-like, *Vitis vinifera*, XP_002271743; ZmHK9, *Zea mays*, AFW87654. **c** Schematic structures of N-terminus of MHZ1 and its homologues. The coiled-coil domain and PAS domain are predicted by SMART (http://smart.embl-heidelberg.de/).

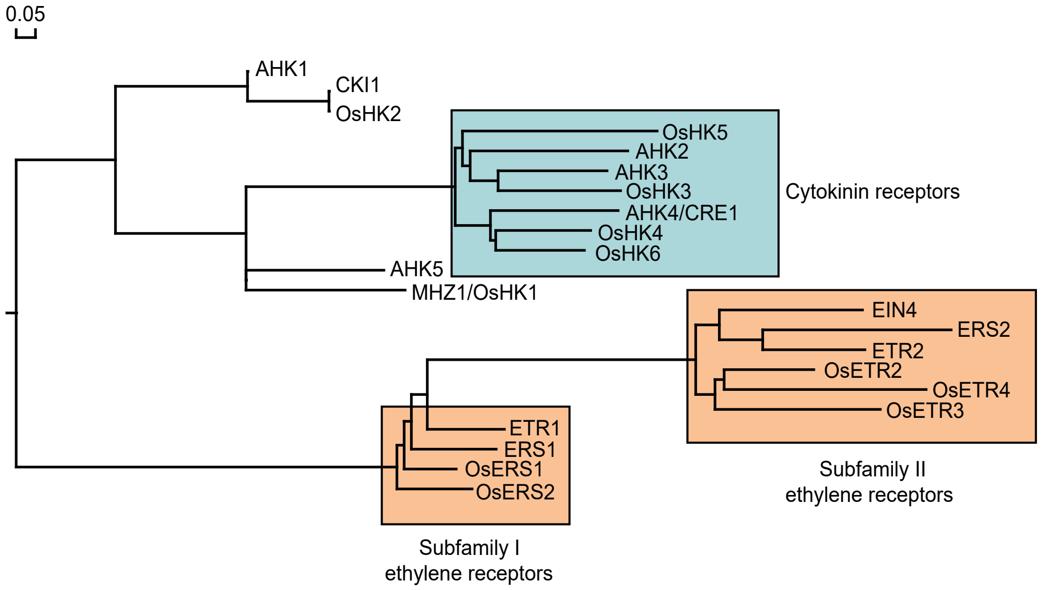

Supplementary Fig. 3 Phylogenetic analysis for histidine kinases in rice and *Arabidopsis*. The phylogenetic tree was generated by DNAMAN with maximum likelihood method. The rooted line distance indicates phylogenetic distance. Proteins include MHZ1/OsHK5, ethylene receptors, cytokinin receptors and other histidine kinases. AHK1, AT2G17820; AHK2, AT5G35750; AHK3, AT1G27320; AHK4, AT2G01830; AHK5, AT5G10720; CKI1, AT2G47430; ETR1, AT1G66340; ETR2, AT3G23150; ERS1, AT2G40940; ERS2, AT1G04310; EIN4, AT3G04580; MHZ1/OsHK1, Os06g44410; OsHK2, Os06g08450; OsHK3, Os01g69920; OsHK4, Os03g50860; OsHK5, Os10g21810; OsHK6, Os02g50480; OsETR2, Os04g08740; OsETR3, Os02g57530; OsETR4, Os07g15540; OsERS1, Os03g49500; OsERS2, Os05g06320.

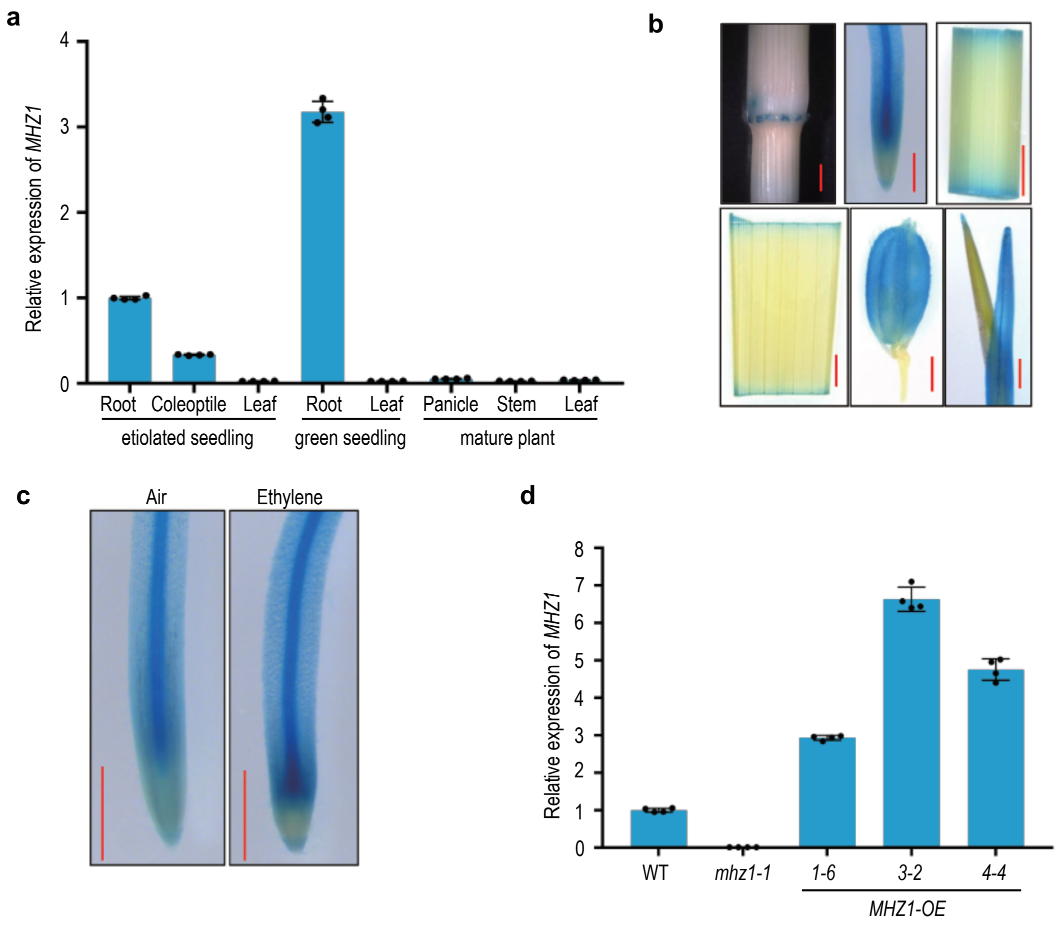

Supplementary Fig. 4 *MHZ1* gene expression and GUS staining analysis. **a** Tissue-specific expression of *MHZ1*. Different organs and tissues of three-day-old etiolated seedlings, two-week-old green seedlings and mature plants were collected for RNA extraction and qPCR analysis. Data are means ± SD, *n* = 4. **b** *MHZ1* promoter activity in different tissues as revealed by promoter-GUS analysis. GUS staining is observed at initiation sites of adventitious roots at nodes, and in root, stem, leaf, grain hull and coleoptile. The red bars indicate 500 μm. **c** *MHZ1* promoter-GUS analysis in response to ethylene. Two-day-old etiolated seedlings were treated with 10 ppm ethylene for 4 h. The root tips were observed under stereomicroscope (Leica, M165 FC). The red bars indicate 500 μm. **d** Expression of *MHZ1* in *MHZ1-OE* lines as revealed by qPCR. Data are means ± SD, *n* = 4. Source data are provided as a Source Data file.

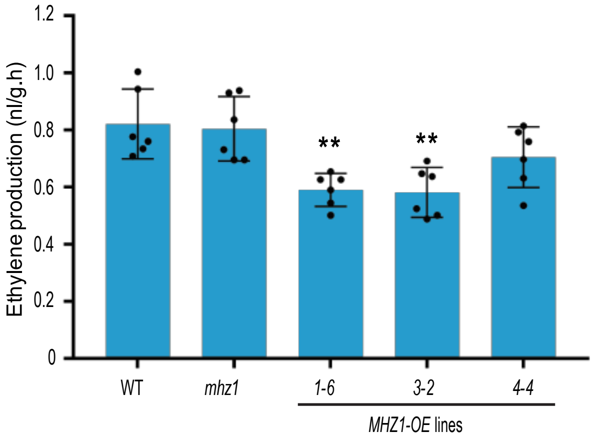

Supplementary Fig. 5 Ethylene production of etiolated seedlings of WT, *mhz1* mutant and *MHZ1-OE* transgenic lines. Seedlings were grown in 40-mL-uncapped vials for 7 d at 28°C under darkness. Vials were then sealed with a rubber syringe cap for 17 h before 1 mL of headspace of each vial was measured using gas chromatography. Values are means ±SD from six biological replicates (**$P < 0.01$; Student's t test; compared with WT) (GC-2014; Shimadzu). Source data are provided as a Source Data file.

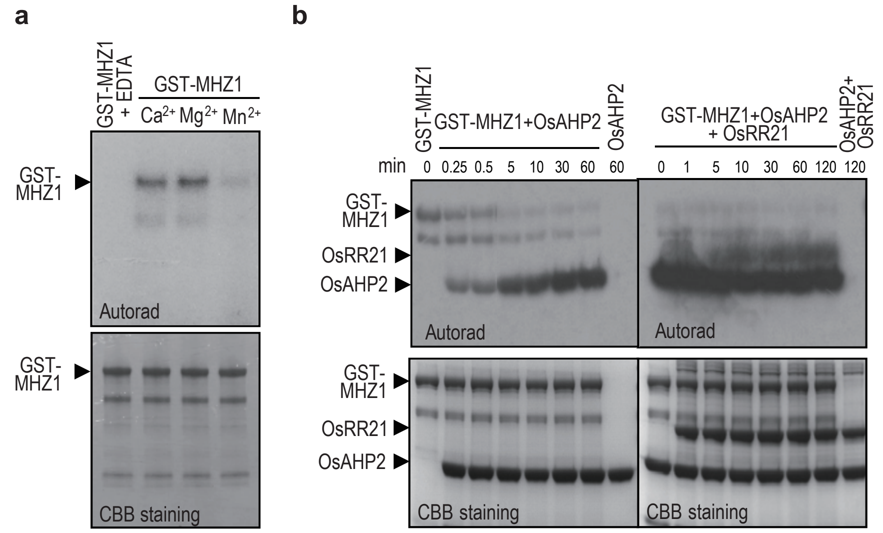

Supplementary Fig. 6 Cation dependence of MHZ1 kinase activity and the phosphorelay. **a** Cation dependence of GST-MHZ1 kinase activity at a physiological level of ATP (0.5 mM). **b** Time course of the phosphorelay from MHZ1 to OsAHP2 (left panel) and further to OsRR21 (right panel). After the reaction in the left panel was finished, OsRR21 protein was added and incubated for various times in the right panel.

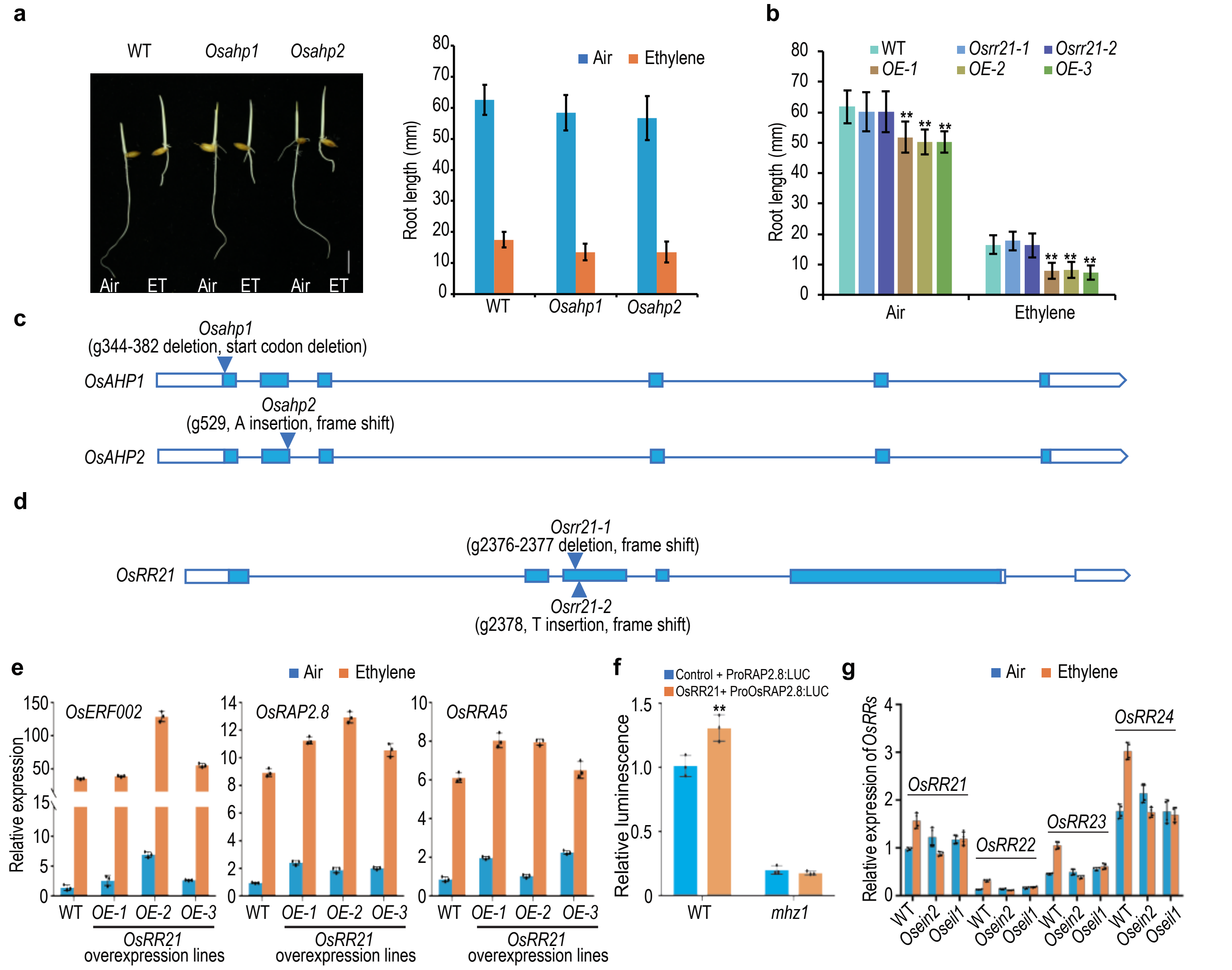

Supplementary Fig. 7 Two-component elements are involved in root ethylene responses of rice. **a** Ethylene response of *Osahp1* and *Osahp2* single mutants. Mutants were generated by CRISPR/Cas9. Mutation sites are listed in **c**. Etiolated seedlings were treated with 10 ppm ethylene or air for 2.5 d. Bars indicate 10 mm. Data are means ± SD, $n > 30$. **b** Root length quantification of *OsRR21* mutants and overexpression lines in fig. 3k. Two mutant lines *Osrr21-1* and *Osrr21-2* were generated by CRISPR/Cas9. Mutation sites are listed in **d**. *OsRR21* overexpresion lines (*OE-1*, *-2*, *-3*) were generated by transgenic approach. Etiolated seedlings were treated with 10 ppm ethylene or air for 2.5 d. Bars indicate 10 mm. Data are means ± SD, $n > 30$ (**$P < 0.01$, compared with WT; Student's *t* test). **e** Ethylene induction of *OsERF002*, *OsRAP2.8* and *OsRRA5* expression was enhanced in *OsRR21-OE* lines as revealed by qPCR. Data are means ± SD, $n = 3$. **f** Activation of OsRAP2.8 promoter activity by OsRR21. Constructs of ProOsRAP2.8:LUC and Pro35S:OsRR21 were cotransformed into protoplasts of WT and *mhz1*. Relative luminescence was detected. Data are means ± SD, $n = 3$ (**$P < 0.01$; Student's *t* test). **g** Ethylene-induced expression of *OsRRs* is dependent on OsEIN2 and OsEIL1 as revealed by qPCR. Two-day-old etiolated seedlings were treated with air or 10 ppm ethylene for 8 h. Data are means ± SD, $n = 4$ (**$P < 0.01$; Student's *t* test). Source data are provided as a Source Data file.

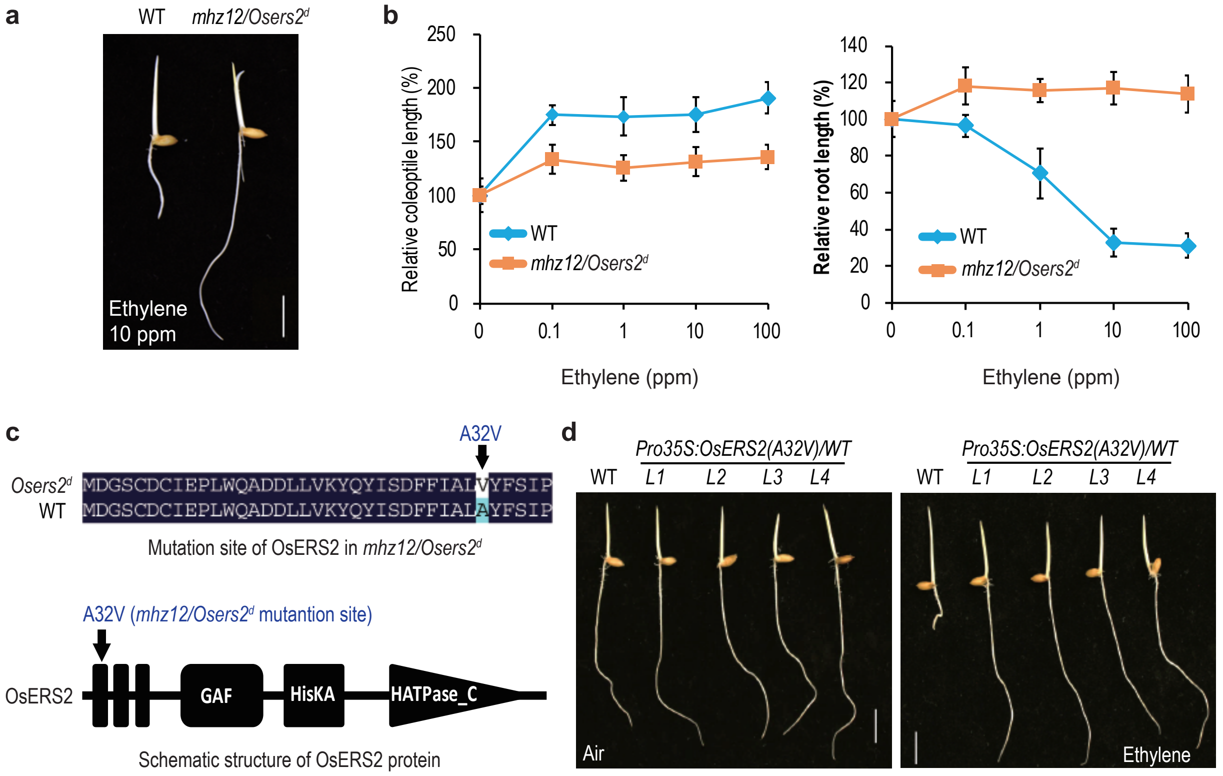

Supplementary Fig. 8 Identification of OsERS2 gain-of-function mutant *mhz12/ Osers2ᵈ*. **a** Ethylene response of *mhz12/Osers2ᵈ* mutant. WT and *mhz12/Osers2ᵈ* etiolated seedlings were grown in darkness under 10 ppm ethylene. Bar indicates 10 mm. **b** Ethylene dose-dependent curves of coleptile and root length of WT and *mhz12/Osers2ᵈ* mutant. Etiolated seedlings were treated with various concentrations of ethylene for 2.5 d. Coleoptile (Left) and root lengths (Right) are means ± SD, *n* > 30. **c** Mutation site of *OsERS2* in *mhz12/Osers2ᵈ* mutant (Above) and the mutated transmembrane domain of OsERS2 in *mhz12/Osers2ᵈ* mutant (Below). **d** Overexpressing OsERS2(A32V) in WT mimics the ethylene insensitive phenotype of *mhz12/Osers2ᵈ*. Transgenic lines over-expressing *OsERS2(A32V)* were generated by tansformation of wild type rice with Agrobacterium strain EHA105 carrying Pro35S:OsERS2(A32V) vector. Etiolated seedlings were grown in darkness with 10 ppm ethylene for 2.5 d. Bars indicate 10 mm. Source data are provided as a Source Data file.

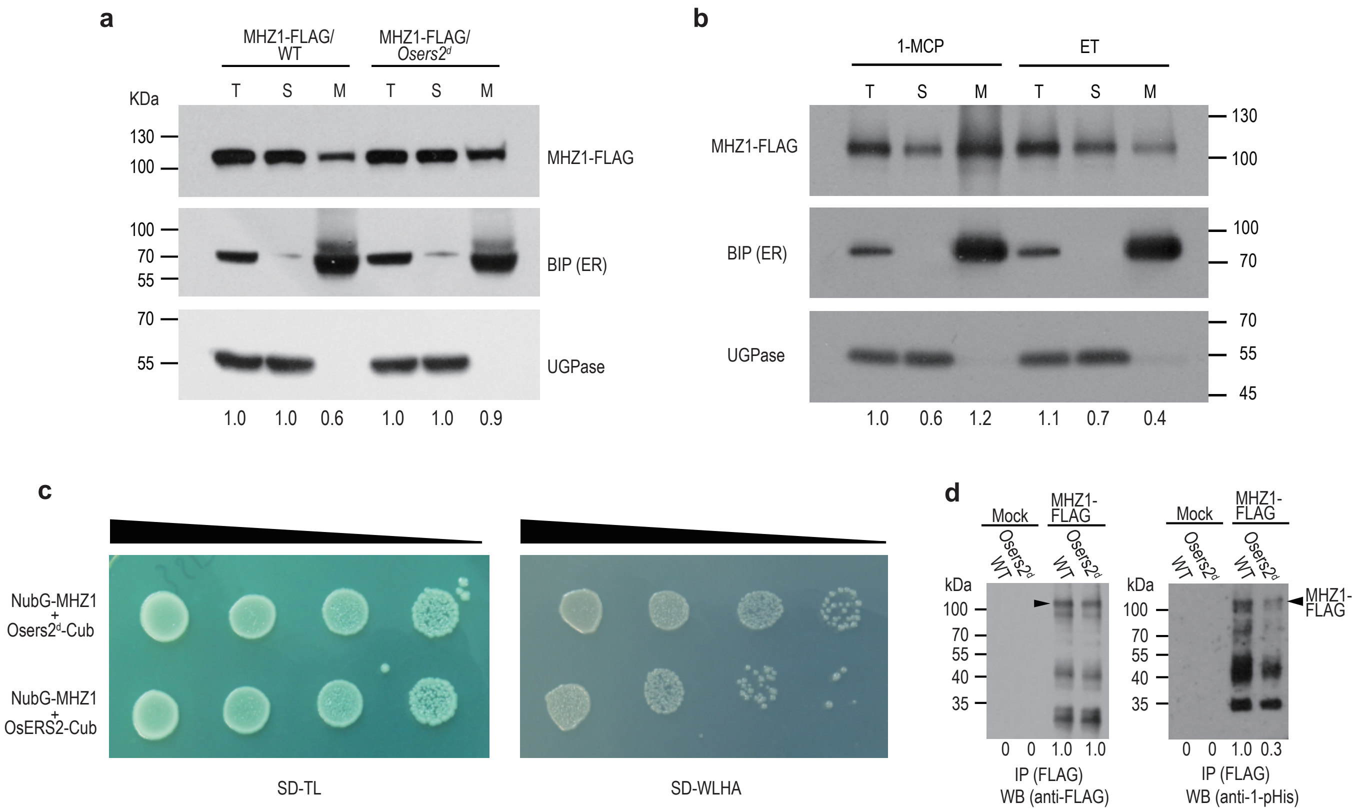

Supplementary Fig. 9 Membrane association of MHZ1 is strengthened by Osers2^{d} (**a**) and 1-MCP treatment (**b**). **a** Vector carring MHZ1-FLAG was transfered into protoplasts of WT and *Osers2^{d}* mutant. Equal amounts of total protein (T), soluble protein (S), and microsomal membranes (M) were immunoblotted for MHZ1, BIP (ER marker), and UGPase (cytoplasm marker). The values at the bottom indicate relative levels of MHZ1-FLAG. **b** Seedlings of *MHZ1OE 4-4* were treated with 1-MCP or ET for 1 h. Equal amounts of total protein (T), soluble protein (S), and microsomal membranes (M) were immunoblotted for MHZ1, BIP (ER marker), and UGPase (cytoplasm marker). Values at the bottom indicate relative levels of MHZ1-FLAG. **c** MHZ1 has a stronger interaction with Osers2^{d} compared with OsERS2 in the Split-ubiquitin Y2H system. Serial decimal dilutions of yeast cells were grown on synthetic complete medium without Leu and Trp (Left) and on synthetic complete medium without His, Leu, Trp and Ade (Right). **d** Histidine phosphorylation of MHZ1 is suppressed in *Osers2^{d}* mutant compared with that in WT. To perform the assay, vector carrying MHZ1-FLAG was transformed into protoplasts of WT and *Osers2^{d}*. MHZ-FLAG protein was immunopreciptated with anti-FLAG affinity gel and immunoblotted with anti-FLAG or anti-1-pHis (Millipore, MABS1330) antibodies. The values at the bottom indicate relative phosphorylation levels of MHZ1. Source data are provided as a Source Data file.

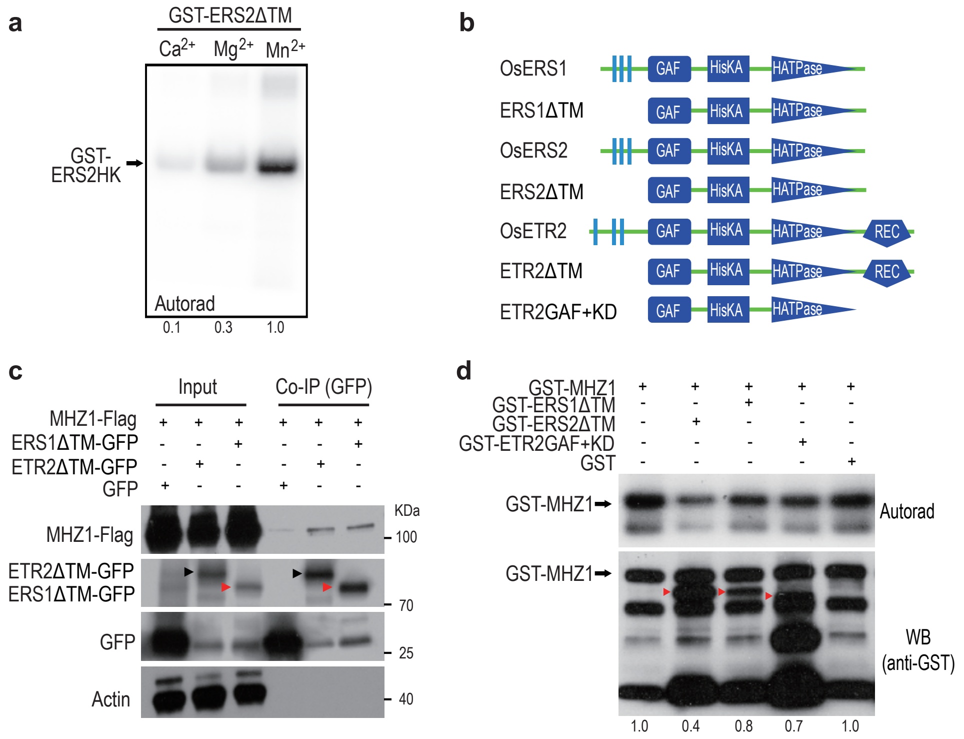

Supplementary Fig. 10 Kinase activity of OsERS2 and the inhibition of MHZ1 kinase activity by different ethylene receptors. **a** Autophosphorylation activity of OsERS2 with different cations. Each reaction contains 2 μg of GST-ERS2ΔTM protein. Values at the bottom indicate relative phosphorylation levels of GST-OsERS2HK. **b** Schematic structures of rice ethylene receptors and their truncated versions. **c** Co-IP assays for interaction of MHZ1 and OsERS1, OsETR2. Constructs containing MHZ1-FLAG and ERS1ΔTM or ETR2ΔTM-GFP were cotransformed into rice protoplasts. Total proteins were immunoprecipitated with GFP-Trap and immunoblotted with anti-GFP, anti-FLAG, and anti-Actin antibodies. **d** OsERS1 and OsETR2 inhibited the kinase activity of MHZ1. GST-ERS1ΔTM and GST-ETR2GAF+KD recombinant proteins were added to the autophosphorylation systems of MHZ1 before the phosphorylation reaction began. GST protein was uesd as a control. The values at the bottom indicate relative phosphorylation levels of GST-MHZ1. Source data are provided as a Source Data file.

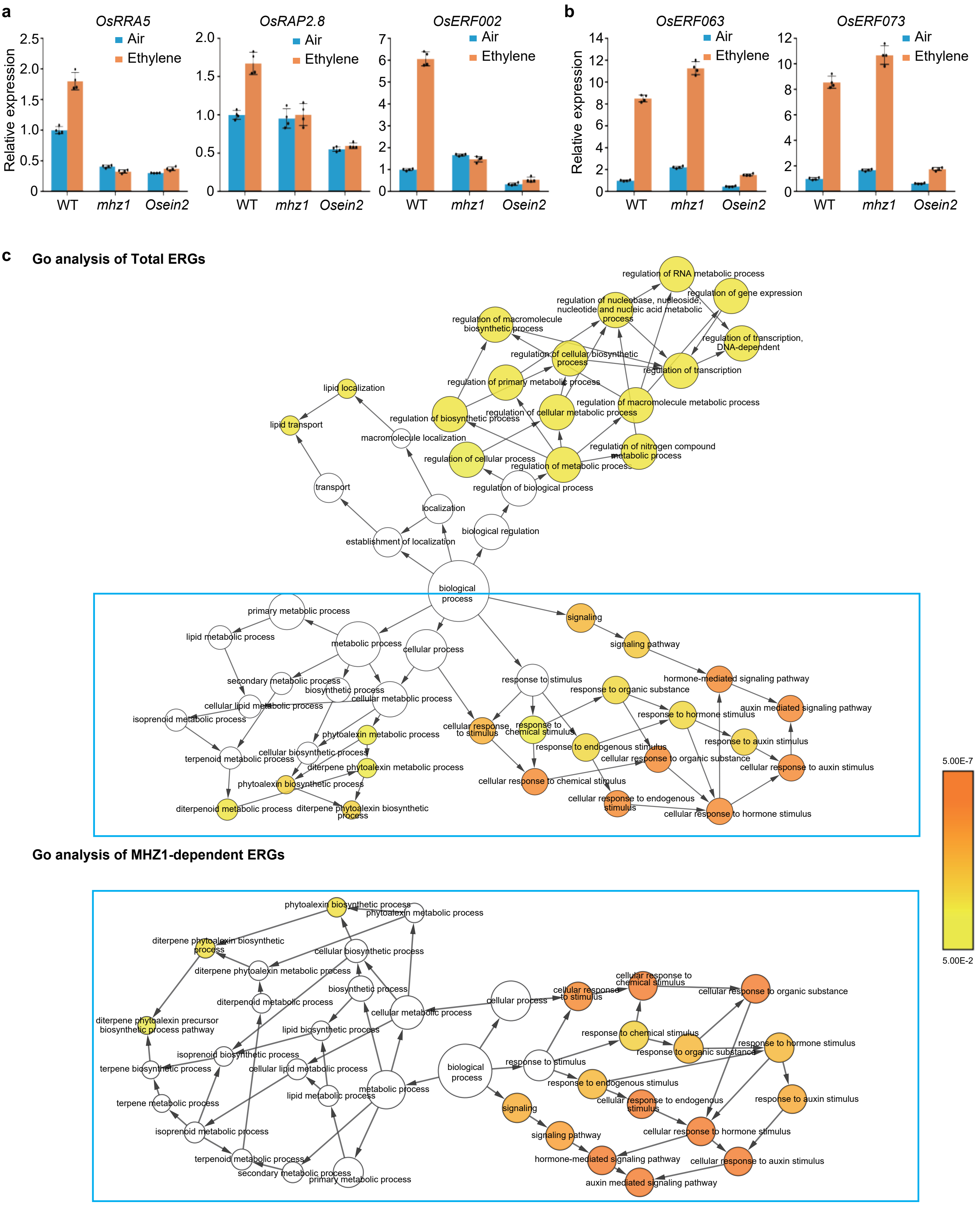

Supplementary Fig. 11 a MHZ1-dependent ethylene-responsive genes. **b** MHZ1-independent ethylene-responsive genes. Data are means ± SD, *n* = 4. 2-d-old etiolated seedlings were treated with air or 10 ppm ethylene for 3 h. **c** GO analysis of total ERGs (ethylene-response genes) and MHZ1-dependent ERGs using BiNGO. Blue boxes indicate the overlapping biological processes.

a

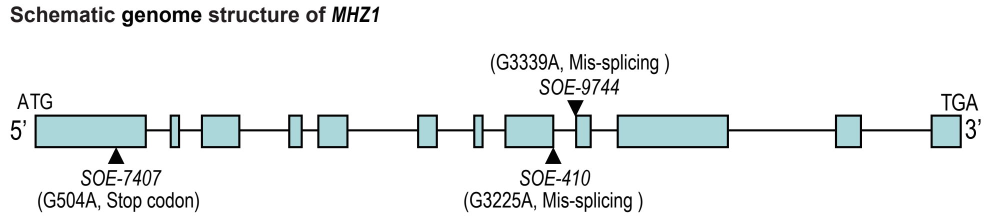

b

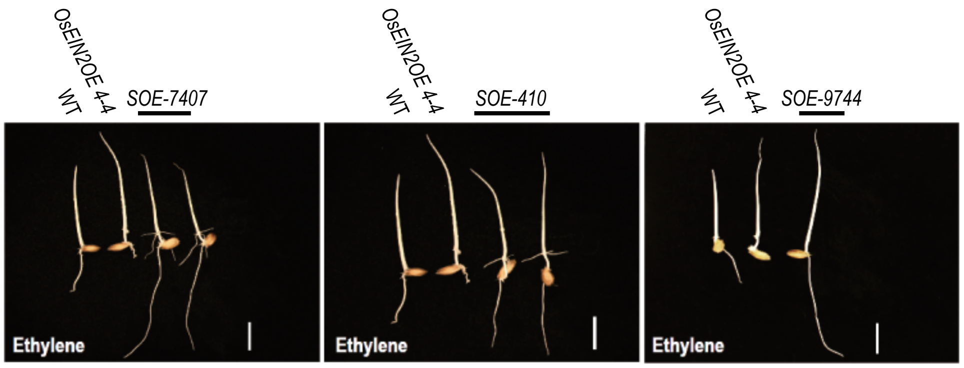

Supplementary Fig. 12 Identification of different *mhz1* alleles by screening for *Supperssors of OsEIN2* (*SOE*). **a** Mutation sites of *MHZ1* in *SOE* lines *7407*, *410* and *9744*. The screening for *OsEIN2* suppressors was carried out by large scale EMS mutagenesis of *OsEIN2* over-expression line *OsEIN2OE-44*. Subsequently, lines of the mutation progeny with altered root ethylene response compared with *OsEIN2OE-44* were selected. After sequencing of the candidate genes, the *MHZ1* was identified to harbor the mutation sites in suppressors including *SOE-7407*,*410* and *9744*. **b** Ethylene response of *SOE* lines *7407*, *410* and *9744*. Seedlings were grown in darkness under 10 ppm ethylene treatment. Bars indicate 10 mm.

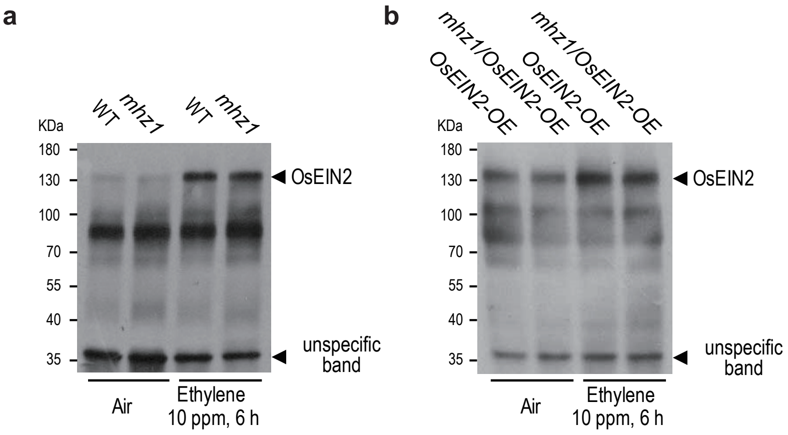

Supplementary Fig. 13 *mhz1* does not affect the stability of OsEIN2 protein. **a** Ethylene-induced OsEIN2 accumulation in *mhz1* roots is identical to that of WT. **b** Ethylene-induced OsEIN2 accumulation in *mhz1/OsEIN2-OE* roots is identical to that of *OsEIN2-OE* line. For (a) and (b), seedlings of different lines were grown in dark for 2 d before treated with air or 10 ppm ethylene for 6 h. Total proteins of roots were immunoblotted for OsEIN2. The unspecific bands served as internal control.

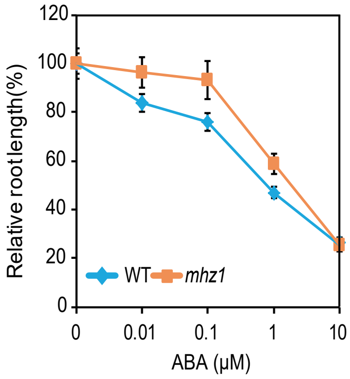

Supplementary Fig. 14 Reponse of *mhz1* mutant to ABA. Etiolated seedlings of *mhz1* mutant were treated with various concentrations of ABA for 3 d. Relative root lengths are means ± SD, $n > 20$. Stock solution of ABA was prepared in ethanol and diluted into solutions of different concentrations with water. Equivalent volumes of ethanol were added to the control. Source data are provided as a Source Data file.

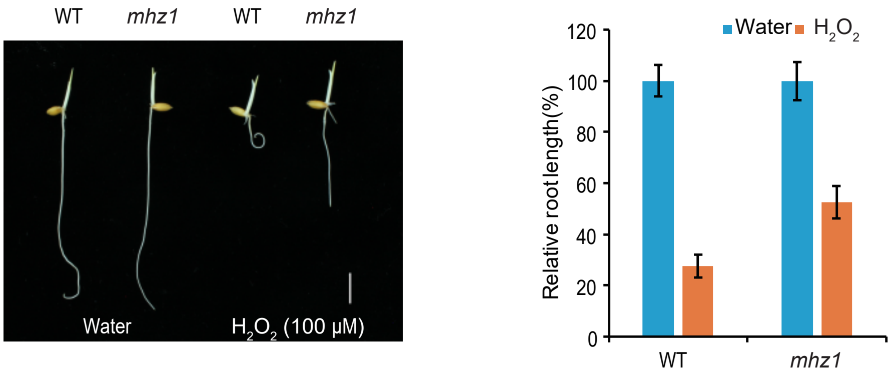

Supplementary Fig. 15 *mhz1* mutant is slightly insensitive to H_2O_2. Etiolated seedlings of WT and *mhz1* mutant were grown in water or 100 μM of H_2O_2 for 3 d. Relative root lengths are means ± SD, $n > 20$. Source data are provided as a Source Data file.

Supplementary Table 1 Primers used in this study.

**Kinase activity assay**		
MHZ1 (365-968aa)	gtcgactcactggggaggcaaatact	gaattccgagcaaaacaaatgctggc
MHZ1-KD (kinase domain)	gaattccgagcaaaacaaatgctggc	gtcgactcaaattttgcaaggcagaacaa
OsPHP1	gaattcatggattattctaatttgcg	gtcgacttacatgacaggcctagtgg
OsAHP1	ggatccatggcggccgccgcgctgac	gtcgacttaatgtttagggtaacaagctt
OsAHP2	gaattcatggcggccgccgctctcc	ctcgagttattgctgcttgggatcataag
OsPHP2	gaattcatggagtattcaaatttgcg	gtcgacttacttccttgagctcactg
OsRR21 (receiver domain)	gaattcatggcgccggtggaggatgg	gtcgactcattgccagatgttcttcaact
OsRR26 (receiver domain)	gaattcatggacgccaccgccttccc	gtcgactcactgccaaatattgcggagct
OsERS2ΔTM	gaattcatggatttgttgaatgtgaagtt	gtcgactacgcttgattggtagcgaa
OsERS2GAF	gaattcgacaggcacaccattctgcg	gtcgactcacatggactcttccaaaatgg
OsERS2KD	gaattcgcccgtaatgattttcttgct	gtcgactcactcaggcatgccaagtttcac
OsERS1ΔTM	gtcgactcttgctgagcgtgaaaacaa	gcggccgctcatatacttctctgataccgag
ETR2GAF+KD	ggatccgatcgccacacgatcctgt	gtcgactcacagctggaactgaagggca
***MHZ1* overexpression**		
MHZ1	ggatccatgggggacgaatacctggct	gtcgacctggggaggcaaatactgcag
3K-promoter	cctgcaggttcaccttagtaggacttcttcac	tctagactttctcaatattaagcgcttcagt
**Identification of genomic complementary transgenic lines**		
Actin1	tccatcttggcatctctcag	gtaccctcatcaggcatctg
NPT II	atggggattgaacaagatggatt	tcagaagaactcgtcaagaagg
MHZ1	ggatccatgggggacgaatacctggct	gtcgacctggggaggcaaatactgcag
T-DNA insertion	cctctttcaggggttcttagcatgg	cgatggctgtgtagaagtactcgc
**Real-time PCR**		
OsERF002	gcagtacgtggaccagatgatc	ctcgatcagagttcttcctcac
OsRAP2.8	gagctcctatgctgccatgt	ggcactatggggatggaagg
OsRRA5	tgggctcggaacctaatgtg	acgacattatcaccaccggg
MHZ1	gccagaaatggatggtctacaag	gtcgacctggggaggcaaatactgcag
OsActin2	ttatggttgggatgggaca	agcacggcttgaatagcg
OsRR21	accctgggtccagttcatct	cggcttggaatgcaagtacc
***mhz1-1* identification**		
MHZ1	ggatccatgggggacgaatacctggct	gtcgacctggggaggcaaatactgcag
***osetr2* mutant identification**		
V-2715LB	ctagagtcgagaattcagtaca	
OsETR2B-F	gcaaaggcttgtaatggatg	
OsETR2B-R	gtctccgactggatgacgaact	
***osers2* mutant identification**		

PR142	attgcgctcgcttacttctc	
PR143	agaatggtgtgcctgtcaag	
PR155	agttttcgcgatccagactg	

***OsRR21* overexpression**

OsRR21	ggatccatggcgccggtggaggatgg	gtcgactcacatctgtccactaaatc

OsEIL1 binding and activation of *MHZ1* promoter activity

MHZ1p-222	ttgtccaactgatttgatacatttaatttattcaattgga	tccaattgaataaattaaatgtatcaaatcagttggacaa
MHZ1p-452	gtgttttttcttggttacatcaattcaccaaagctgaac	gttcagctttggtgaattgatgtaaccaagaaaaaacac
OsEIL1-N	cgggatccatgggaggtggtctggtgat	gggaattctcagccgccggtaggtgccgga
35S-OsEIL1	cgggatccatgatgggaggtggtctggtgat	gctctagaggtgctctttctttaaacgctacaa
MHZ1 2K-promoter	gaattcatccatatgtcatagaccgc	ggatccctttctcaatattaagcgcttcagt

Split-ubiquitin Y2H assay for interaction of MHZ1 and OsERS2.

ERS2-STE	ttaattaaggccgcctcggccatggatggatcatgtgattgc	cgataagcttgatatcgaatttacgcttgattggtagcgaa
M1-pRR3	acggcccgggaaaaaacatgtatgggggacgaatacctgg	cggtatcgataagcttgatattcactggggaggcaaatact

Pull down assay

GST-ETR2ΔTM	gaattcatggatttgttgaatgtgaagtt	gtcgactacgcttgattggtagcgaa

Co-IP assay

ERS1ΔTM-GFP	ggcgcgccactagtggatccatgttgctgagcgtgaaaacaa	catcccgggagcggtacctatacttctctgataccgag
ERS2ΔTM-GFP	acgcgtcgacatggatttgttgaatgtgaagtt	ggggtacctacgcttgattggtagcgaa
ERS2GAF-GFP	acgcgtcgacatgaggcacaccattctgcgaa	ggggtacccatggactcttccaaaatgg
ERS2KD-GFP	acgcgtcgacatggcccgtaatgattttcttgc	ggggtaccctcaggcatgccaagtttc
ETR2ΔTM-GFP	ggcgcgccactagtggatccatggatcgccacacgatcctgt	catcccgggagcggtacccagctggaactgaagggca
