# Supplementary material for: Histidine kinase MHZ1/OsHK1 interacts with ethylene receptors to regulate root growth in rice
Source: Nat Commun. 2020 Jan 24;11:518. doi: 10.1038/s41467-020-14313-0 (PMC6981129; doi:10.1038/s41467-020-14313-0)
Supplement: Supplementary file 3 — Description of Additional Supplementary Files [file 41467_2020_14313_MOESM3_ESM.pdf]

Description of Additional Supplementary Files

File Name: Supplementary Data 1
Description: RNA-seq analysis of ethylene-related genes in WT, mhz1, Oseil1 and Osein2.
