# Supplementary material for: Histidine kinase MHZ1/OsHK1 interacts with ethylene receptors to regulate root growth in rice
Source: Nat Commun. 2020 Jan 24;11:518. doi: 10.1038/s41467-020-14313-0 (PMC6981129; doi:10.1038/s41467-020-14313-0)
Supplement: Supplementary file 5 — Reporting Summary [file 41467_2020_14313_MOESM5_ESM.pdf]

nature research

Corresponding author(s): Jin-Song Zhang

Reporting Summary

Nature Research wishes to improve the reproducibility of the work that we publish. This form provides structure for consistency and transparency in reporting. For further information on Nature Research policies, see Authors & Referees and the Editorial Policy Checklist.

Statistical parameters

When statistical analyses are reported, confirm that the following items are present in the relevant location (e.g. figure legend, table legend, main text, or Methods section).

n/a	Confirmed	
☐	☒	The exact sample size (*n*) for each experimental group/condition, given as a discrete number and unit of measurement
☐	☒	An indication of whether measurements were taken from distinct samples or whether the same sample was measured repeatedly
☐	☒	The statistical test(s) used AND whether they are one- or two-sided<br>*Only common tests should be described solely by name; describe more complex techniques in the Methods section.*
☒	☐	A description of all covariates tested
☒	☐	A description of any assumptions or corrections, such as tests of normality and adjustment for multiple comparisons
☐	☒	A full description of the statistics including central tendency (e.g. means) or other basic estimates (e.g. regression coefficient) AND variation (e.g. standard deviation) or associated estimates of uncertainty (e.g. confidence intervals)
☒	☐	For null hypothesis testing, the test statistic (e.g. *F*, *t*, *r*) with confidence intervals, effect sizes, degrees of freedom and *P* value noted<br>*Give P values as exact values whenever suitable.*
☒	☐	For Bayesian analysis, information on the choice of priors and Markov chain Monte Carlo settings
☒	☐	For hierarchical and complex designs, identification of the appropriate level for tests and full reporting of outcomes
☒	☐	Estimates of effect sizes (e.g. Cohen's *d*, Pearson's *r*), indicating how they were calculated
☐	☒	Clearly defined error bars<br>*State explicitly what error bars represent (e.g. SD, SE, CI)*

Our web collection on statistics for biologists may be useful.

Software and code

Policy information about availability of computer code

Data collection	Western blot grey value quantification was carried out using imageJ.
Data analysis	For RNA-seq analysis, clean data was remapped to rice genome by TopHat2.0 and analyzed with Cufflinks software.

For manuscripts utilizing custom algorithms or software that are central to the research but not yet described in published literature, software must be made available to editors/reviewers upon request. We strongly encourage code deposition in a community repository (e.g. GitHub). See the Nature Research guidelines for submitting code & software for further information.

Data

Policy information about availability of data

All manuscripts must include a data availability statement. This statement should provide the following information, where applicable:
- Accession codes, unique identifiers, or web links for publicly available datasets
- A list of figures that have associated raw data
- A description of any restrictions on data availability

The authors declare that all data supporting the findings of this study are available within the manuscript and the Supplementary Files or are available from the corresponding authors upon request. The source data underlying Figs 1a, 1e, 1f, 2a-c, 3i, 4a-c, 4g-m, 5b, 5e and Supplementary Figs 4a, 4d, 5, 7a, 7db, 7e-g, 8b, 9a, 9b, 10c, 10d, 11a, 11b, 14 and 15 are provided as a Source Data file. RNA sequence data reported in this paper have been deposited in the Genome Sequence

Archive (Genomics, Proteomics & Bioinformatics 2017) in BIG Data Center (Nucleic Acids Res 2019), Beijing Institute of Genomics (BIG), Chinese Academy of Sciences, under accession numbers CRA002197 that is publicly accessible at https://bigd.big.ac.cn/gsa.

Field-specific reporting

Please select the best fit for your research. If you are not sure, read the appropriate sections before making your selection.

[x] Life sciences [ ] Behavioural & social sciences [ ] Ecological, evolutionary & environmental sciences

For a reference copy of the document with all sections, see nature.com/authors/policies/ReportingSummary-flat.pdf

Life sciences study design

All studies must disclose on these points even when the disclosure is negative.

Sample size	The sample size was determined according to the reports in the related research subjects. For coleoptile and root lengths measurements, at least 30 seedlings of each line was used. For MHZ1 promoter activity analysis, three biological replicates were performed. For ethylene production analysis, six biological replicates were performed for each sample. For RNA-seq analysis, three biological replicates were performed for each sample. No statistical methods were used to predetermine sample sizes.
Data exclusions	No data were excluded from the analyses.
Replication	All attempts at replication were successful.
Randomization	The experiments were performed to compare the phenotypes or gene expression levels between wild type, mutants and transgenic lines, therefore, sample allocation is not relevant to this study.
Blinding	The experiments were performed to compare the phenotypes or gene expression levels between wild type, mutants and transgenic lines, therefore, blinding is not relevant to this study.

Reporting for specific materials, systems and methods

Materials & experimental systems

n/a	Involved in the study
[ ]	[x] Unique biological materials
[ ]	[x] Antibodies
[x]	[ ] Eukaryotic cell lines
[x]	[ ] Palaeontology
[x]	[ ] Animals and other organisms
[x]	[ ] Human research participants

Methods

n/a	Involved in the study
[x]	[ ] ChIP-seq
[x]	[ ] Flow cytometry
[x]	[ ] MRI-based neuroimaging

Unique biological materials

Policy information about availability of materials

Obtaining unique materials	All the mhz mutants and the overexpression lines used in this study are available from the corresponding authors upon request.

Antibodies

Antibodies used	anti-flag antibody, MBL, Cat. # M185-3L, clone FLA-1, Lot # 010; anti-GST antibody, Abmart, Cat. # M20007M, clone 12G8, Lot # 253986; anti-GFP antibody, Abmart, Cat. # M20004H, clone 7G9, Lot # 294175; anti-MBP antibody, CWBIO, Cat. # CW0288M, clone 6D3, Lot # 01254/40217; anti-1-pHis antibody, Millipore, Cat. # MABS1330, clone SC1-1, Lot # 2912011; anti-UGPase antibody, Agrisera, Cat. # AS05 086, Polyclonal; anti-BiP antibody, Agrisera, Cat. # AS09 481, Polyclonal; anti-Actin antibody, Abmart, Cat. # M20009L, clone 26F7, Lot # 254579. The anti-OsEIN2 antibody was generated by Ma (Ma et al., 2018).
Validation	Application of primary antibodies to detect rice proteins: anti-OsEIN2 (1:10,000), anti-GFP (1:5,000), anti-BiP (1:5,000), anti-UGPase (1:5,000) , anti-FLAG (1:2,000), anti-1-pHis (1:1000), anti-Actin (1:5000). Application of primary antibodies to detect E. coli expressed proteins: anti-GST (1:5000), anti-MBP (1:5000). Primary antibody dilutions were in PBS containing 3% milk and 0.1% Tween 20. For OsEIN2 and 1-pHis detection, the primary antibodies were diluted in Immunoreaction Enhancer Solution I (Toyobo).
